# Human Immunodeficiency Virus, Antiretroviral Therapy and Markers of Lymphatic Filariasis Infection: A Cross-sectional Study in Rural Northern Malawi

**DOI:** 10.1371/journal.pntd.0003825

**Published:** 2015-06-04

**Authors:** Terence Tafatatha, Miriam Taegtmeyer, Bagrey Ngwira, Amos Phiri, Mariot Kondowe, Wilson Piston, Anna Molesworth, Ndoliwe Kayuni, Olivier Koole, Amelia Crampin, John Horton, Neil French

**Affiliations:** 1 Karonga Prevention Study, Karonga District, Malawi; 2 Centre for Neglected Tropical Diseases, Liverpool School of Tropical Medicine, Liverpool, United Kingdom; 3 Department of International Public Health, Liverpool School of Tropical Medicine, Liverpool, United Kingdom; 4 London School of Hygiene and Tropical Medicine, London, United Kingdom; 5 Tropical Projects, Hitchin, United Kingdom; 6 Institute of Infection and Global Health, University of Liverpool, Liverpool, United Kingdom; National Institutes of Health, UNITED STATES

## Abstract

**Background:**

Lymphatic filariasis (LF) and human immunodeficiency virus (HIV) are major public health problems. Individuals may be co-infected, raising the possibility of important interactions between these two pathogens with consequences for LF elimination through annual mass drug administration (MDA).

**Methodology and Principal Findings:**

We analysed circulating filarial antigenaemia (CFA) by HIV infection status among adults in two sites in northern Malawi, a region endemic for both LF and HIV. Stored blood samples and data from two geographically separate studies were used: one a recruitment phase of a clinical trial of anti-filarial agent dosing regimens, and the other a whole population annual HIV sero-survey. In study one, 1,851 consecutive adult volunteers were screened for HIV and LF infection. CFA prevalence was 25.4% (43/169) in HIV-positive and 23.6% (351/1487) in HIV-negative participants (p=0.57). Geometric mean CFA concentrations were 859 and 1660 antigen units per ml of blood (Ag/ml) respectively, geometric mean ratio (GMR) 0.85, 95%CI 0.49-1.50. In 7,863 adults in study two, CFA prevalence was 20.9% (86/411) in HIV-positive and 24.0% (1789/7452) in HIV–negative participants (p=0.15). Geometric mean CFA concentrations were 630 and 839 Ag/ml respectively (GMR 0.75, 95%CI 0.60-0.94). In the HIV-positive group, antiretroviral therapy (ART) use was associated with a lower CFA prevalence, 12.7% (18/142) vs. 25.3% (67/265), (OR 0.43, 95%CI 0.24-0.76). Prevalence of CFA decreased with duration of ART use, 15.2% 0-1 year (n=59), 13.6% >1-2 years (n=44), 10.0% >2-3 years (n=30) and 0% >3-4 years treatment (n=9), p<0.01 χ^2^ for linear trend.

**Conclusions/Significance:**

In this large cross-sectional study of two distinct LF-exposed populations, there is no evidence that HIV infection has an impact on LF epidemiology that will interfere with LF control measures. A significant association of ART use with lower CFA prevalence merits further investigation to understand this apparent beneficial impact of ART.

## Author Summary

Lymphatic filariasis (LF) and HIV are both major public health problems worldwide and where they co-exist have the potential to interact. The main strategy for LF elimination is annual mass drug administration (MDA). A particular concern is whether HIV, through its impact on the immune system, will interfere with the effectiveness of this approach to control and eliminate LF. We report findings from cross-sectional studies in two separate populations in northern Malawi where both HIV and LF are common. One group (1,851 individuals) were studied at enrolment into a trial of anti-LF treatments, whilst the other study used samples stored from adult participants in a whole population HIV survey (7,863 individuals). Between 5–10% of the study participants were HIV-positive and 24% were LF-infected. We found no evidence that LF infection was more or less common in HIV-positive adults in either population. However, we identified robust evidence that antiretroviral therapy use was associated with lower LF prevalence rates. We have no evidence to suggest HIV will have a detrimental effect on LF control. On the contrary, the evidence suggests that antiretroviral therapy may have beneficial effects and merits further careful evaluation of the anti-filarial properties of these compounds.

## Introduction

Human immunodeficiency virus (HIV) and parasitic infections affect widely overlapping populations in sub-Saharan Africa. Of the estimated 35 million people infected with HIV worldwide at the end of 2013, about 70% were from sub-Saharan Africa [1]. Parasitic infections, including lymphatic filariasis (LF) are also widespread in sub-Saharan Africa, raising the possibility of clinically significant interactions between the two pathogens. It has been suggested that HIV and parasitic co-infections may have bidirectional deleterious interactions by affecting susceptibility to HIV, impacting on HIV progression and potentially worsening clinical outcomes of filarial infection [2]. Previous in-vitro studies have shown helminth infections to increase susceptibility of peripheral blood mononuclear cells to HIV infection [3]. In addition deworming can result in increases in CD4+ cells and reduction in plasma HIV-1 RNA concentrations [4]. Derangements in the immune response associated with HIV-infection might also be expected to alter susceptibility to, or complications from, filarial infection or other helminths such as *Strongyloides* [5]. To date, there are few studies that have investigated LF and HIV co-infection and to our knowledge, none have been on a large population scale. A cross-sectional study of 907 adults undertaken in Tanga region of Tanzania reported increased circulating filarial antigen (CFA) concentration in HIV-positive persons [6], although a further evaluation of this group of individuals did not support any association between HIV and *Wuchereria bancrofti* infection [7]. Similarly, in urban southern India, no quantitative difference in *W*. *bancrofti* CFA levels by HIV status was found in a study of 432 HIV-positive and 99 HIV-negative patients [8].

Malawi embarked on a programme of mass drug administration (MDA) for LF control and elimination in 2009 [9]. Concerns that the programme may be less effective in areas of high HIV and LF prevalence prompted this study in Karonga, a district in the northern region of Malawi which was known to be highly endemic for LF infection [10]. Karonga is bordered by Lake Malawi to the east, the Songwe river to the north (which also forms the boundary with Tanzania), and by the Nyika plateau and escarpment to the west and south. The population is rural and dependent on subsistence agriculture including rice growing and fishing from the lake.

Two previous studies had been undertaken by co-authors in the district and both had serum samples stored with approval for later testing. The first was the recruitment phase of a randomised controlled clinical trial that investigated alternative schedules and dosing regimens of ivermectin and albendazole use in MDA programmes (study 1). Findings from this clinical trial are reported elsewhere [11]. The second study was nested within a comprehensive population-based HIV survey which enabled a longitudinal assessment of CFA in a whole adult population (study 2). In this paper we report the prevalence and relationship of LF and HIV infections from these two studies.

## Methods

Study 1 used samples and data collected as part of the screening phase of a clinical trial of the effectiveness of increasing the dose and frequency of albendazole and ivermectin as antifilarial agents for clearing LF microfilaraemia (clinical trials registration number NCT01213576) [11]. It was undertaken between January 2009 to March 2012 in the northern portion of Karonga district along the Tanzanian border and Songwe river delta. Villages in this area had previously been shown to have a high prevalence of filarial antigenaemia and chronic manifestations of LF [10]. No mass treatment interventions had been undertaken in the area at the time the study was started.

Enrolment to the clinical trial required individuals to have a microfilarial count of >80 microfilariae per ml of blood. Consequently a population-based screening process was undertaken to identify suitable participants for enrolment in the therapeutic trial. The estimated total adult population of the target villages was 36,643 [12]. Sensitization meetings were held with community members, the Traditional Authority (TA) and all the village headmen and their aides who are administratively responsible for the study area. At these meetings the aims and procedures of the study were explained. Following verbal approval by the community leaders, a team of field workers went house-to-house seeking written consent and recruiting individual participants. All households in selected villages were visited in sequence before moving on to the next village. This screening phase was planned to continue until 120 eligible individuals with appropriate microfilarial levels were recruited into the trial. However, screening and recruitment into this study was discontinued following the rollout of the albendazole/ivermectin national MDA programme in the study area as further recruitment into the clinical trial became impractical. At the end of the recruitment phase individuals from 16 villages were included. For analysis purposes smaller villages were combined in a geographically appropriate way to produce 10 village location categories with suitable numbers of participants.

Individuals were eligible if they provided written informed consent, were residents of the area and aged between 18 and 55. At each home visit, the study was introduced and explained to all members and individual participants were asked to provide written informed consent by signing (or thumb printing if they were illiterate) the informed consent forms that were translated in the local language (Tumbuka). A questionnaire was administered to capture personal details. Eligible individuals were screened for CFA by the immunochromatographic (ICT) card test and for HIV by trained counsellors following the national HIV rapid testing algorithm. CFA positive individuals were asked to provide a night blood sample between 22:00 and 02:00 hours when a 5ml sodium citrate sample was collected for microfilaria counting and later stored in the project laboratory archive at -20°C. All individuals who were CFA positive but declined to participate in the clinical trial or did not meet the eligibility criteria for the trial were offered standard dose antifilarial therapy with albendazole and ivermectin. Individuals who were HIV positive were referred for HIV treatment and care. Assessment of HIV clinical stage, CD4 count and viral load were not performed on these individuals as a part of the study protocol.

Study 2 used samples and data from an annual whole adult population survey. Repeated rounds of data and sample collection spanned the time periods before and after the introduction of MDA. It was undertaken in the Karonga Health and Demographic Surveillance Site (KHDSS) and nested within a comprehensive population-based HIV sero-survey [13]. The KHDSS area is mapped and divided into geographically defined clusters of 20–30 households which are further aggregated into 21 geographically distinct reporting groups. The KHDSS was established between August 2002 and August 2004 to serve as a sampling frame for on-going epidemiological and clinical studies. Unlike study 1, data from study 2 included detailed socio-demographic information allowing for inclusion of these factors in statistical analysis. Since its establishment, the initial population of the KHDSS has been under continuous demographic surveillance. In addition, between September 2007 and October 2011 four annual HIV serological surveys have been conducted in all individuals aged 15 or more years using rapid point-of-care HIV tests on finger-prick whole blood samples [14]. Community sensitisation meetings to explain the aims and procedures of the study were held in each village and were followed by house-to-house visits by counsellors to recruit participants. The counsellors were trained and certified by Malawi Ministry of Health staff to perform HIV counselling and testing and referral using standard procedures [15]. Written informed consent was obtained as in study 1. In addition, all consenting adults were asked to provide a 5ml blood sample for quality control and storage for further laboratory analysis including for other diseases of importance in Karonga district. Plasma samples from consenting participants were stored in the project laboratory archive at -20°C. Samples from the first surveillance round, which took place between September 2007 and October 2008, prior to MDA introduction, were included in our study. Viral load, CD4 counts and clinical staging were not measured as a part of the survey.

### Common laboratory methods for both studies

The ICT card test (Binax, Portland, ME) [16] was only used as the screening test for LF infection in study 1. This is a portable rapid point-of-care test suitable for screening in non-laboratory settings and was used in accordance with manufacturer’s instructions. Microfilaria counting was done on ICT card test positive individuals in study 1 by the nucleopore membrane filtration technique [17]. This involves filtering a measured volume of venous blood through a 5μM pore size nucleopore filter. After filtration, the filter is removed, placed on a glass slide and mounted on a light microscope for examination and counting of microfilariae. Filarial antigenaemia was quantified in all plasma samples in both study 1 and study 2 by means of the Og4C3 antigen-capture ELISA (TropBio, Australia) [18]. Samples were analysed in accordance with the manufacturer’s instructions and expressed as antigen units per ml of blood (Ag/ml) based on control samples supplied with the ELISA kits. Samples with CFA concentration more than 128Ag/ml were considered clear positives for CFA. Samples with CFA concentration of 32Ag/ml and below were considered negatives. Samples with a titre between 32 and 128Ag/ml were considered indeterminate.

In both studies HIV testing used whole blood rapid diagnostic tests according to Malawian national guidelines [15] with demonstrated high accuracy in community settings in Karonga [14]. The initial screening test was with Determine TM HIV-1/2 (Abbott Japan Co Ltd, Japan) and confirmatory testing was done with UniGold TM HIV-1/2 (Trinity Biotech PLC, Ireland). Samples with a non-reactive screening test were considered negative and those with a reactive screening and confirmatory test were considered positive. Where the screening and confirmatory tests were discordant, a tie breaker using a third rapid test, (SD Bioline, Korea) was used.

### Common data management and statistical methods for both studies

Study forms were checked, coded, double entered and verified using Microsoft Access software. Statistical analysis was done in Stata 12 software (StataCorp, Texas, USA). Continuous variables were log transformed prior to analysis to achieve an approximate normal distribution. Linear regression was used for crude and adjusted analyses with results expressed as geometric mean ratios (GMR) and their 95% confidence intervals. The association of age, gender and CFA status with HIV positivity was estimated using χ^2^ tests for crude analyses and a logistic regression model for adjusted Odds Ratios (OR). In a risk factor analysis for CFA positivity, logistic regression was used to estimate crude and adjusted ORs. Variables were retained in the model if significant associations were identified in the unadjusted estimates. Geographic identifiers for groups of survey villages were also incorporated in the models to adjust for geographic confounding as previous surveys had indicated heterogeneity of CFA prevalence across the region [19]. Rather than a binary variable, HIV was treated as a categorical variable in the model with the HIV—negative group and two HIV-positive groups based on ART use at the time of blood sampling. A sub-group analysis to investigate the effect of cotrimoxazole and duration of ART use on CFA prevalence was performed on the HIV-positive group only. Logistic regression models were used to estimate odds ratios. Difference in CFA prevalence with increased use of ART was investigated with a χ^2^ test for linear trend with odds ratios derived from a 2xn table. Adjusted odds ratios were estimated with logistic regression.

### Ethics

The National Health Sciences Research Committee of the Malawi Ministry of Health (protocol numbers 495 and 419) and the Ethical Committee of the London School of Hygiene and Tropical Medicine (protocol numbers 5344 and 5081) gave ethical clearance for both studies 1 and 2. Study participants in both studies were consented for storage and later testing of samples at the time of enrolment. This covered testing for HIV and other diseases of local significance. The National Health Sciences Research Committee (protocol number 908) and the Ethical Committee of the Liverpool School of Tropical Medicine (protocol number 11.77) approved the additional analysis conducted on stored samples.

## Results

The overall population of Karonga district during the study period was 272,789 [12] with the KHDSS population accounting for 33,500. HIV prevalence in the KHDSS in the 2007–2008 survey year was measured at 7.4% but estimated to be 10.4% when adjusted for non-testing by those who already knew they were HIV-positive [20]. At baseline 54.8% of those aged 15 years or more reported previous HIV testing.

### Study 1

**Fig 1:**
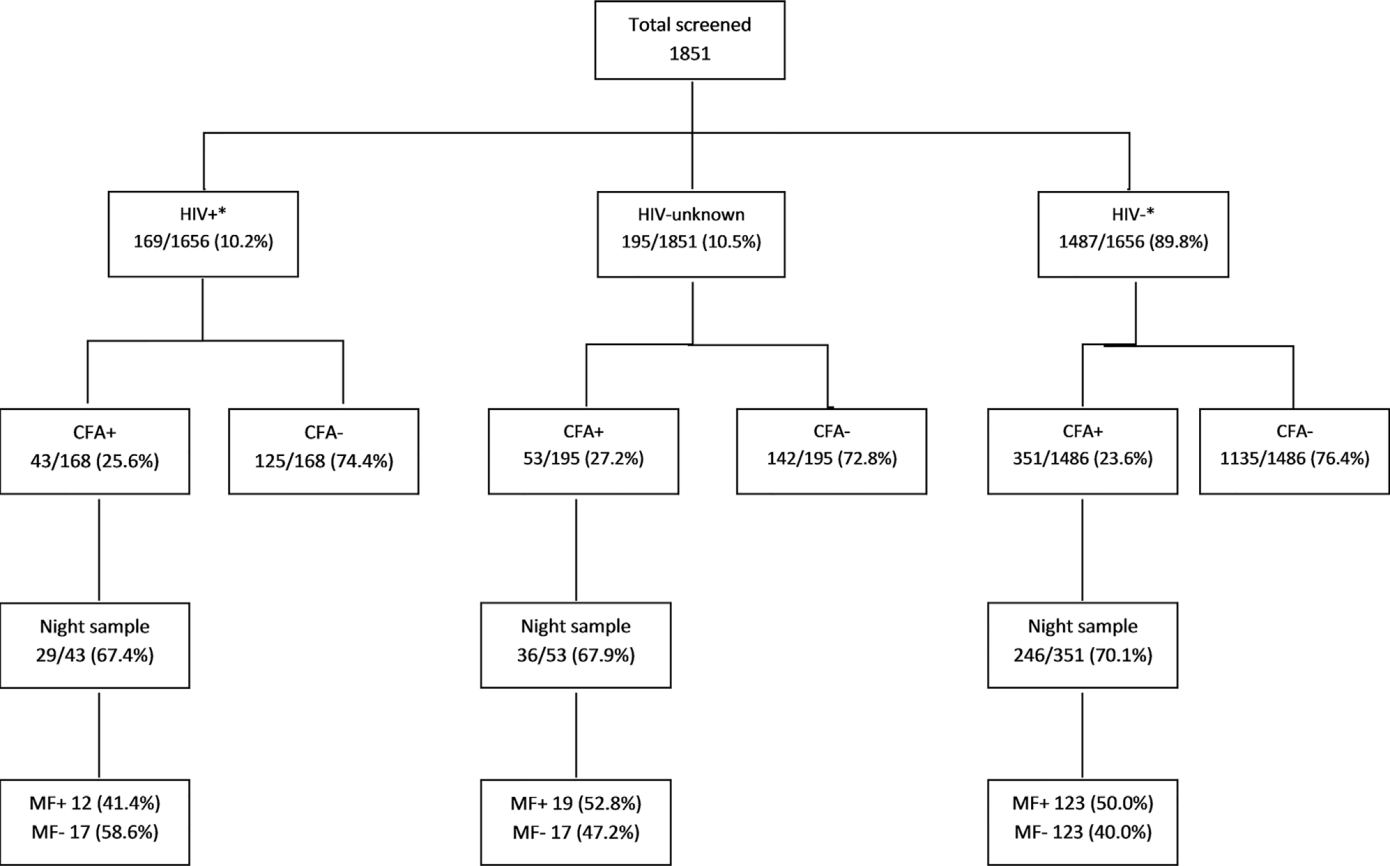
Study 1 flow chart detailing the breakdown of individuals by HIV status, circulating filarial antigen (CFA) status by immunochromatographic card (ICT) test and microfilarial counts. *2 individual had an invalid ICT test. MF—microfilaria.

From the estimated total of 36,643 adults of the target villages, 1,851 individuals were eligible and consented to participate. Of these 1,851 individuals screened for LF antigen by the ICT card test, 447 (24.2%) were CFA positive (Fig 1). A total of 1,656 individuals accepted HIV testing and 169 (10.2%) of these were HIV-positive. HIV-positive individuals tended to be older (Table 1). CFA positivity was present in 43 (25.6%) of HIV-positive and 351 (23.6%) of HIV-negative (crude OR 1.11, 95% CI 0.77–1.60) with an LF/HIV co-infection prevalence rate of 2.6%. CFA positivity did not differ by HIV infection status (Table 1). There was heterogeneity in the prevalence of CFA by village location, median 24.4%, range 15.7–33.3% (Pearson χ^2^ 22.7, p<0.01, 9 degrees of freedom). Data on the use of antiretroviral and cotrimoxazole was incomplete in the context of this study.

**Table 1:** Baseline characteristics of Study 1 participants by HIV status.

**A**				
**Characteristic**	**HIV-positive (n = 169)**	**HIV-negative (n = 1487)**	**OR (95% CI^†^)**	**Adjusted OR (95% CI)#**
**Age group**				
**18–29 years**	43 (25.4%)	727 (48.9%)	-	-
**30–39 years**	68 (40.2%)	406 (27.3%)	**2.83 (1.90–4.23)**	**2.83 (1.90–4.23)**
**40 years and above**	58 (34.3%)	354 (23.8%)	**2.77 (1.83–4.19)**	**2.69 (1.77–4.08)**
**Sex**				
**Male**	66 (39.0%)	618 (41.6%)	-	-
**Female**	103 (61.0%)	869 (58.4%)	1.11 (0.80–1.54)	1.14 (0.82–1.59)
**CFA status**				
**Negative**	125 (74.4%)	1135 (76.4%)	-	-
**Positive**	43 (25.4%)	351 (23.6%)	1.11 (0.77–1.60)	1.13 (0.78–1.65)
**MF status**				
**Positive**	17 (58.6%)	123 (50.0%)	-	-
**Negative**	12 (41.4%)	123 (50.0%)	0.71 (0.32–1.54)	0.81 (0.35–1.85)
**B**				
**Characteristic**	**HIV-positive**	**HIV-negative**	**GMR (95% CI)**	**Adjusted GMR (95% CI)** ^**#**^
**CFA GMC Ag/ml (95% CI)**	859 (231–3193)	1660 (1198–2302)	0.85 (0.49–1.50)	0.91 (0.55–1.51)
**C**				
**Characteristic**	**HIV-positive**	**HIV-negative**	**P value**	
**MF count, median (IQR), mf/ml**	0 (0–22)	1 (0–93)	0.13	

**A)** The association of age group, gender and circulating filarial antigen (CFA) with HIV positivity. Adjusted Odds Ratio (OR) derived from logistic regression model. **B)** Geometric mean concentration (GMC) of CFA in those CFA positive. Geometric mean ratio (GMR) derived from linear regression model. **C)** Microfilaria (MF) count in those CFA positive expressed as median and interquartile range, difference assessed by rank sum testing owing to the skewed nature of data.

^†^CI—confidence interval;

^#^ adjusted for age, sex and village location

Microfilaria counting was done in the 311 (69.6%) LF antigen positive individuals who were eligible and gave consent for night blood sampling. The remainder either refused or left before follow up. HIV prevalence in those lost to follow up was broadly similar to those sampled (10.3% vs. 9.3% respectively, χ^2^ test p = 0.90). Microfilariae were present in 49.5% of the 311 sampled individuals. Microfilarial detection and levels did not differ by HIV infection status (Fig 1 and Table 1).

Of 311 stored baseline night blood plasma samples, 290 (93.2%) were CFA positive using the Og4C3 antigen-capture ELISA. CFA was positive in 26 (89.7%) of HIV-positive individuals, 231 (93.9%) of the HIV-negative individuals and 33 (97.1%) of HIV-unknown individuals respectively (p = 0.47). The geometric mean CFA concentration levels by HIV status were 859 and 1660 for HIV-positive and HIV-negative respectively (GMR 0.85, 95% CI 0.49–1.50). CFA and MF counts showed reasonable positive correlation (Pearson correlation coefficient r = 0.56, p<0.01).

### Study 2

The total eligible population of 15 year olds and older in the KDHSS at the baseline survey was 11,756. From this group, 7,863 (66.9%) underwent HIV testing and consented to storage of their blood sample. Of these, 1,875 (23.9%) individuals were CFA positive by the Og4C3 ELISA. HIV infection was identified in 411 (5.2%) participants. HIV-positive adults tended to be older and more likely to be female (Table 2). CFA positivity was present in 86 (20.9%) of HIV-positive and 1789 (24.0%) of HIV-negative (crude OR 0.84, 95% CI 0.66–1.07) with an HIV/LF co-infection prevalence rate of 4.6%. In the female participants, CFA positivity was present in 17.8% of the HIV-positive and 19.8% of the HIV-negative (OR 0.88, 95% CI 0.64–1.21) and in the male participants, 26.8% of the HIV-positive and 29.4% of the HIV-negative (OR 0.88, 95% CI 0.60–1.28) respectively. Geometric mean CFA concentration was lower in the HIV-positive individuals by 25% although this association was weakened when adjusted for age and sex (Table 2).

**Table 2:** Baseline characteristics of study 2 participants by HIV status.

**A**				
**Characteristic**	**HIV-positive (n = 411)** ^**#**^	**HIV-negative (n = 7452)** ^**#**^	**OR (95% CI^†^)**	Adjusted OR (95% CI)*
**Age group**				
**15–29 years**	77 (18.7%)	3793 (50.9%)	-	-
**30–39 years**	159 (38.7%)	1480 (19.9%)	**5.29 (4.00–6.99)**	**5.35 (4.04–7.07)**
**40 years and above**	175 (42.6%)	2179 (29.2%)	**3.96 (3.01–5.20)**	**4.02 (3.06–5.29)**
**Sex**				
**Female**	269 (65.5%)	4201 (56.4%)	-	-
**Male**	142 (34.5%)	3251 (43.6%)	**0.68 (0.55–0.84)**	**0.72 (0.58–0.89)**
**CFA status**				
**Negative**	325 (79.1%)	5663 (75.9%)	-	-
**Positive**	86 (20.9%)	1789 (24.0%)	0.84 (0.66–1.07)	0.86 (0.67–1.10)
**B**				
**Characteristic**	**HIV-positive**	**HIV-negative**	**GMR (95% CI)**	Adjusted GMR (95% CI) *
**CFA GMC, Ag/ml (95% CI)**	630 (511–778)	839 (799–882)	**0.75 (0.60–0.94)**	0.62 (0.38–1.02)

**A)** The association of age group, gender and circulating filarial antigen (CFA) with HIV positivity. Adjusted Odds Ratio (OR) derived from logistic regression model. **B)** Geometric mean concentration (GMC) of CFA in those CFA positive, Geometric Mean Ratio (GMR) derived from linear regression model.

^#^ 2 HIV-positive and 26 HIV-negative with incomplete data;

^**†**^CI—confidence interval;

* Adjusted for age, sex, and reporting group

Several risk factors were associated with an increased prevalence of CFA (Table 3). These included male gender, age between 30 and 39 and lower quality housing, whilst decreased CFA prevalence was associated with higher levels of education (p<0.01, χ^2^ for linear trend) the availability of piped tap water or the use of lake water and the use of antiretrovirals. Bed net ownership was high, however ownership or the number of nets owned in the household was not associated with CFA prevalence. Individuals were found in all 21 reporting groups, with a median of 314 participants (range 129–820). There was considerable heterogeneity in the prevalence of CFA by reporting group, median 23.2% range 5.7–37.2% (Pearson χ^2^ 354.7, p<0.01, 20 degrees of freedom).

**Table 3:** The association of circulating filarial antigenaemia (CFA) prevalence with HIV and antiretroviral therapy (ART) status and major potential confounding socio-demographic characteristics in the 7,863 study 2 participants.

Characteristic	N (%)	CFA prevalence (%)	OR (95% CI^#^)	aOR (95% CI)
**HIV and ART status**				
HIV-negative	7452 (94.8)	1789 (24.0)	Ref*	Ref
HIV-positive—no ART	265 (3.4)	67 (25.3)	1.07 (0.81–1.42)	1.20 (0.90–1.62)
HIV-positive—ART	142 (1.8)	18 (12.7)	**0.46 (0.28–0.76)**	**0.50 (0.30–0.84)**
**Gender**				
Male	3392 (43.1)	995 (29.3)	**1.69 (1.52–1.88)**	**1.77 (1.59–1.98)**
Female	4470 (56.9)	880 (19.7)	Ref	Ref
**Age group**				
Age 15–29 years	3870 (49.2)	896 (23.2)	Ref	Ref
Age 30–39 years	1639 (20.9)	440 (26.7)	**1.22 (1.07–1.39)**	**1.24 (1.08–1.43)**
Age 40 years and above	2354 (29.9)	539 (22.9)	0.99 (0.87–1.11)	0.95 (0.84–1.09)
**Mosquito net ownership**				
0	197 (2.5)	46 (23.4)	Ref	-
1	945 (12.0)	254 (26.9)	1.21 (0.84–1.73)	-
2	1817 (23.1)	461 (25.4)	1.12 (0.79–1.58)	-
3	1835 (23.3)	430 (23.4)	1.00 (0.71–1.42)	-
≥4	2854 (36.3)	640 (22.4)	0.95 (0.67–1.34)	-
unknown	215 (2.7)	-	-	-
**Educational achievement**				
Nil	277 (3.6)	83 (30.0)	Ref	Ref
Primary	5493 (71.3)	1379 (25.1)	0.78(0.60–1.02)	**0.71 (0.54–0.93)**
Secondary	1886 (24.5)	377 (20.0)	**0.58 (0.44–0.77)**	**0.55 (0.41–0.75)**
Tertiary	45 (0.6)	8 (17.8)	0.51 (0.23–1.13)	0.52 (0.22–1.19)
Unknown	2 (0.0)	-	-	
**Water supply**				
Bore hole	3755 (47.7)	1037 (27.6)	Ref	Ref
Tap to house	1082 (13.8)	105 (9.7)	**0.28 (0.23–0.35)**	**0.30 (0.24–0.37)**
Shared tap	835 (10.6)	127 (15.2)	**0.47 (0.38–0.57)**	**0.48 (0.39–0.59)**
Covered well	999 (12.7)	303 (30.3)	1.14 (0.98–1.33)	1.16 (0.99–1.35)
Open well	572 (7.3)	167 (29.2)	1.08 (0.89–1.31)	1.06 (0.87–1.30)
Lake	456 (5.8)	106 (23.3)	**0.79 (0.63–1.00)**	**0.77 (0.61–0.97)**
Unknown	11 (0.1)	-	-	-
**Housing type**				
Burnt brick	5750 (73.1)	1328 (23.1)	Ref	
Unburnt brick	678 (8.6)	169 (24.9)	1.11 (0.92–1.33)	1.00 (0.83–1.21)
Mud	1087 (13.8)	299 (27.5)	**1.26 (1.09–1.46)**	1.03 (0.88–1.20)
Grass/bamboo	167 (2.1)	49 (29.3)	1.38 (0.98–1.94)	1.14 (0.81–1.62)
Other	18 (0.2)	2 (11.1)	0.42 (0.10–1.81)	0.43 (0.10–1.91)
Unknown	163 (2.1)	-	-	-

Crude and adjusted Odds Ratios (OR) derived from a logistic regression model. Data on reporting group are not shown in the table but adjusted models include this as a potential confounder along with the other significant variables in the crude analysis.

^#^CI—confidence interval:

*Reference category

**Fig 2:**
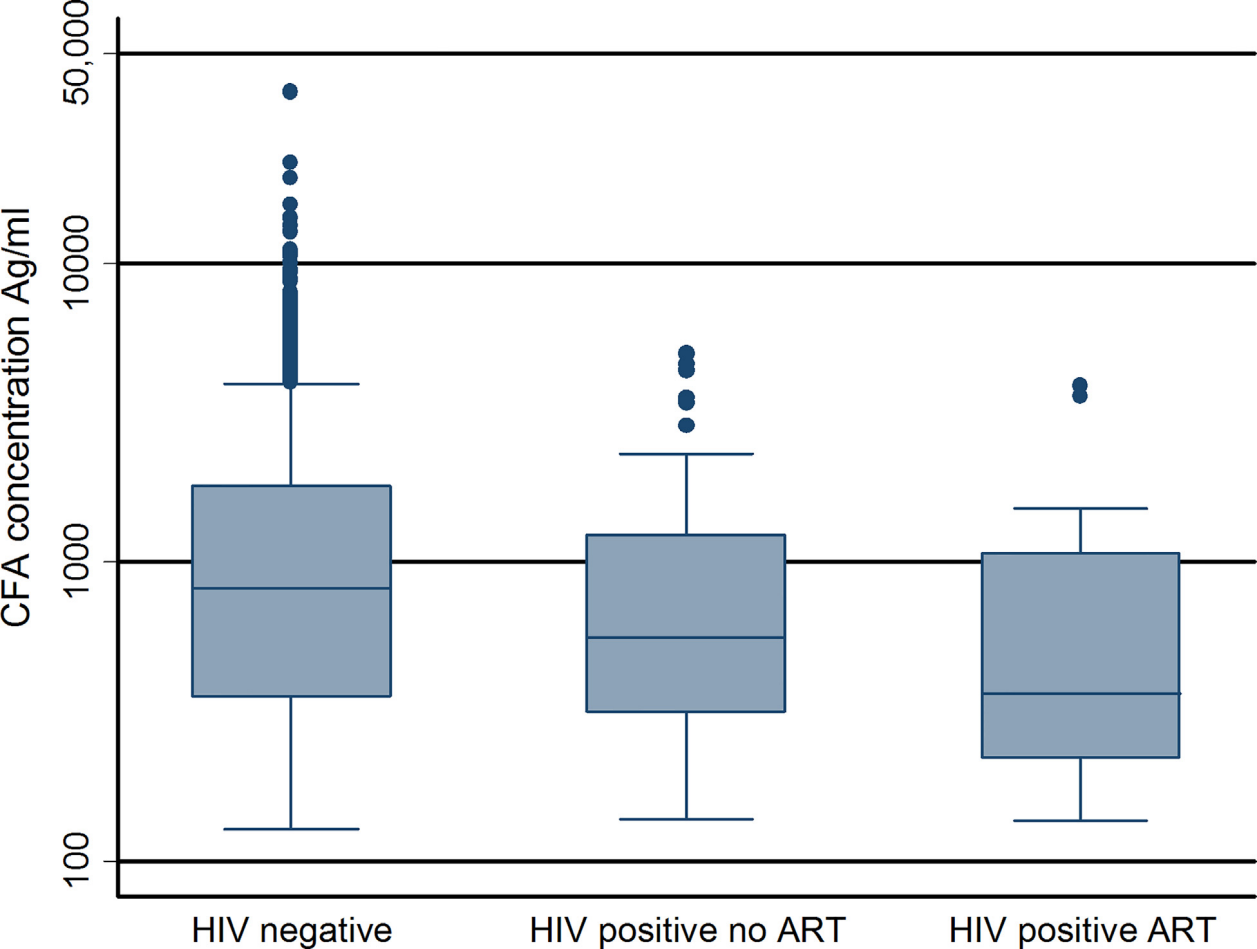
Box plot of Circulating Filarial Antigen (CFA) concentration distribution (on logarithmic scale) by HIV and antiretroviral treatment (ART) status for study 2 participants. The boxes show the median value and the interquartile range. The whiskers include all values within 1.5 times the interquartile range with outliers shown as points. Comparison of CFA concentrations by group HIV- vs. HIV +/ART- (p = 0.22): HIV- vs. HIV+/ART+ (p = 0.05): HIV+/ART- vs. HIV+/ART+ (p = 0.28), derived from a linear regression model adjusted for age, gender and reporting group.

Of the 411 HIV-positive adults, 142 (34.5%) were taking antiretroviral therapy (ART) and 117 (28.5%) were using cotrimoxazole prophylaxis (CTX) with only 4 of the 117 taking CTX without ART at the time of sampling. In 6 of the 411 individuals, information on ART and/or CTX use at the time of sampling was unavailable. ART consisted of Lamivudine, Stavudine and Nevirapine (Triomune-30) in 94% of cases with Zidovudine or Efavirenz substitutions in the remainder. No protease inhibitors were in use. In the HIV-positive group, ART use was associated with a lower prevalence of CFA when compared to those not on ART [12.7% vs. 25.3% (OR 0.43, 95% CI 0.24–0.76)]. Similarly, CTX use was associated with lower CFA prevalence [12.8% vs. 24.1% (OR 0.46, 95% CI 0.25–0.85)]. In a multivariable model incorporating ART and CTX use along with age, sex and geographical location, the adjusted odds ratio for ART use was 0.47 (95% CI 0.17–1.31) and for CTX use 0.92 (95% CI 0.31–2.71). When the ART treated group were further sub-divided by year since treatment started, there was a significant trend to decreased prevalence of CFA with increasing time on treatment; 25.3% no treatment (n = 265), 15.2% year 1 treatment (n = 59), 13.6% year 2 treatment (n = 44), 10.0% year 3 treatment (n = 30) and 0% year 4 treatment (n = 9), (p<0.01 χ^2^ for linear trend). This relationship persisted after adjustment for age, gender and reporting group. In the HIV-positive individuals with detectable CFA, the geometric mean concentration of CFA was not significantly different between those off and on ART, 647 vs. 512 Ag/ml respectively, GMR 1.27, 95% CI 0.76–2.08 (Fig 2), nor did the GMC differ by ART duration category 647, 392, 762, 516 & 0 Ag/ml for no treatment, year 1, 2, 3 & 4 of treatment respectively.

## Discussion

We present data from two separate studies undertaken in Karonga district, northern Malawi. In both studies a high LF and HIV prevalence was measured with HIV co-infection rates of 2.6% and 4.6% among those who were CFA positive and 15 years and older. We found no evidence that HIV is associated with an increased risk of LF infection. Initial findings from study 1, a clinical trial not powered to definitively test the impact of HIV on LF infection, revealed a tendency to lower CFA and microfilarial density in the HIV-positive adults. Subsequent investigation of these parameters in the much larger population sample revealed a tendency to lower CFA prevalence in the HIV-positive group, attributable to significantly lower CFA prevalence in the ART treated sub-group, a finding that persisted following adjustment for key potential confounders and showed a significant trend to lower CFA prevalence with duration of ART use. There was no significant effect of CTX therapy, when analysed in a multivariable model. CFA concentration was also persistently lower in the HIV-positive group although at a level of uncertain clinical or public health significance.

Previous studies have reported divergent findings with some showing an association between LF and HIV infections but these have tended to be small samples and in selected populations [6–8]. In contrast to these studies, our second study had a larger sample taken from a whole population survey, including a high proportion of the at-risk population in an area with high prevalence rates of LF and HIV. The findings in relation to ART use are novel and we are unaware of other studies that have investigated this association. Individuals receiving ART may represent a select group of the HIV-positive population who have better health seeking behaviour, may be more educated, live in better accommodation and/or may live in close proximity to health providers. However, as this work was undertaken in the context of a demographic survey we were able to investigate these potential confounders by adjusting for reported educational status, housing quality, access to clean water and geographic location. The finding of ART associated with a lower CFA prevalence appears robust.

An explanation for these findings remains less clear and merits further work. Residual confounding or an unrecognised selection bias remains possible, but seems unlikely given the highly significant lower CFA prevalence with duration of ART therapy. The crude association of CTX with lower CFA prevalence seems adequately explained by concomitant use of ART, and there is no evidence to support either sulphonamides or trimethoprim, the components of CTX, as effective antifilarial agents. If LF infection adversely impacts on the success of ART therapy, then over time the prevalence of CFA positivity in this group will reduce as the LF/HIV co-infected die. There is no evidence from the Malawi national HIV programme that outcomes from ART treatment are worse in regions of the country endemic for LF compared to those with low LF prevalence. Helminth infections have been linked to increased viral load in non-ART treated individuals [21] but not to evidence of faster HIV progression [22]. Similarly, LF infection had no significant effect on HIV disease progression in a study of *W*. *bancrofti* and HIV coinfections in south India [23]. Altered diagnostic accuracy of the Og4C3 ELISA in the presence of ART has not been reported. ART has been rarely linked to false negative HIV results in children and adults but this is more likely to be due to low levels of virus and/or antibody than a direct inhibitory effect. The reduction in CFA prevalence by ART treatment duration and the antigen capture nature of the Og4C3 ELISA would be difficult to explain by ART inhibition of the assay. Immune reconstitution as a result of ART does not adequately explain our finding either as there is a similar prevalence of CFA in the HIV-negative and the HIV-positive untreated. There is no precedent for immune recovery following ART leaving the immune system in a more competent state than an HIV-negative person. ART treatment is an imprecise proxy marker of duration of HIV infection. If the natural history of LF in the HIV-positive is a steady fall in antigenaemia could this explain the association? We do not have accurate seroconversion dates for the majority of this population so are not able to fully consider this possibility. However with ART use the “natural history” of HIV is dramatically altered and it might be expected that any tendency to lower antigenaemia with time would also be altered and this would be inconsistent with our findings. The most plausible explanation for this finding is a direct filaricidal activity of the major ART agents. We are unaware of any data on the effect of Lamivudine, Stavudine or Nevirapine on helminths. Further evaluation of these molecules as antihelminthics would be appropriate.

Of the other factors associated with CFA positivity all have been reported previously, providing reassurance that the epidemiology of LF disease in Karonga is not unique and results are generalizable to other similar regions. One surprise was the lack of association with bed net ownership. However most households possessed bed nets limiting the power of any comparison, and during this survey we did not specifically ask about usage, or condition of the nets, thus limiting the value of this finding. More detailed evaluation of this will be needed in future work.

Both of our studies had some degree of selection bias, but it is unlikely that this has fundamentally altered our findings. In study 1, we targeted villages known historically to have a high prevalence of LF infection. If participation by HIV-positive individuals was reduced because of perceived stigma associated with an HIV test, we may have had reduced power to identify an association between LF and HIV. However, a similar finding in the much larger study 2 provides consistency. In study 2, we know HIV-positive adults were under-represented. Adults who knew their status from earlier HIV testing studies or through routine service provision in the district, declined participation [20]. However it is difficult to see a mechanism whereby LF co-infection would disproportionately lead to non-participation by HIV-positive adults and in particular ART treated HIV-positive adults thereby obscuring the true association.

More females than males were included in both studies. This may represent the easier access to females at the time of recruitment since females are more likely to be at home. Although we know men are more likely to be infected with LF in this population, we do not think this under-representation has meaningfully affected the LF/HIV association. Sub-group analyses showed similar odds ratios for the LF/HIV association by gender in study 2 suggesting no major effect modification.

The measurement of our exposure (HIV) and outcome endpoints (LF status) were based on accurate and well described tests and we do not believe these have introduced significant bias into the study. We used different tests for assessment of circulating filarial antigen in the two studies with different sensitivities and specificities, the ICT card test with sensitivity and specificity reportedly close to 100% and the Og4C3 ELISA test with 100% sensitivity and specificity of at least 94% [24–26]. There was some disparity between these two tests identified in study 1. This is consistent with previous studies that have reported overall agreement between the ICT and Og3C4 tests but different sensitivities and specificities [24,25]. In study 2, we were not able to assess MF counts due to the use of a stored sample collection. Whilst we cannot categorically rule out an association between MF density and HIV, data from study 1 showed a positive correlation between CFA levels and MF density. Previous studies have also shown a positive correlation between CFA levels and MF density [26,27]. This implies that the CFA relationship will broadly apply to MF counts.

In summary, we did not demonstrate a significant detrimental association between LF and HIV in these studies that will have a negative impact on plans to eliminate lymphatic filariasis. However ART treated adults had significantly lower CFA prevalence, a finding that merits further careful evaluation to exclude an adverse impact of LF on HIV, or the potential of antiretrovirals as molecules with antihelminthic properties.

## Supporting Information

S1 Checklist STROBE Checklist. (DOCX)
